# Axitinib and HDAC Inhibitors Interact to Kill Sarcoma Cells

**DOI:** 10.3389/fonc.2021.723966

**Published:** 2021-09-16

**Authors:** Jane L. Roberts, Laurence Booth, Andrew Poklepovic, Paul Dent

**Affiliations:** ^1^Department of Pharmacology and Toxicology, Virginia Commonwealth University, Richmond, VA, United States; ^2^Department of Biochemistry and Molecular Biology, Virginia Commonwealth University, Richmond, VA, United States; ^3^Department of Medicine, Virginia Commonwealth University, Richmond, VA, United States

**Keywords:** axitinib, HDACi, autophagy, PD-L1, MHCA, sarcoma

## Abstract

We have extended our analyses of HDAC inhibitor biology in sarcoma. The multi-kinase inhibitor axitinib interacted with multiple HDAC inhibitors to kill sarcoma cells. Axitinib and HDAC inhibitors interacted in a greater than additive fashion to inactivate AKT, mTORC1 and mTORC2, and to increase Raptor S722/S792 phosphorylation. Individually, all drugs increased phosphorylation of ATM S1981, AMPKα T172, ULK1 S317 and ATG13 S318 and reduced ULK1 S757 phosphorylation; this correlated with enhanced autophagic flux. Increased phosphorylation of ULK1 S317 and of Raptor S722/S792 required ATM-AMPK signaling. ULK1 S757 is a recognized site for mTORC1 and knock down of either ATM or AMPKα reduced the drug-induced dephosphorylation of this site. Combined exposure of cells to axitinib and an HDAC inhibitor significantly reduced the expression of HDAC1, HDAC2, HDAC3, HDAC4, HDAC6 and HDAC7. No response was observed for HDACs 10 and 11. Knock down of ULK1, Beclin1 or ATG5 prevented the decline in HDAC expression, as did expression of a constitutively active mTOR protein. Axitinib combined with HDAC inhibitors enhanced expression of Class I MHCA and reduced expression of PD-L1 which was recapitulated *via* knock down studies, particularly of HDACs 1 and 3. *In vivo*, axitinib and the HDAC inhibitor entinostat interacted to significantly reduce tumor growth. Collectively our findings support the exploration of axitinib and HDAC inhibitors being developed as a novel sarcoma therapy.

## Introduction

Soft tissue sarcomas (STS) are a rare mesenchymal-derived and diverse group of tumors, with over 50 subtypes. Approximately 12,000 STS cases are diagnosed each year in the USA with approximately 5,000 deaths. Treatment of sarcomas involves surgery, radiotherapy, and chemotherapy e.g., pazopanib. Patients who present with metastatic disease have a survival just over 2 years. New drugs and regimens are urgently needed to improve outcomes in this disease.

We recently demonstrated that the multi-kinase inhibitor pazopanib interacted with HDAC inhibitors *in vitro* and *in vivo* to kill sarcoma cells and suppress tumor growth (1, 2). One component of the evolutionary resistance mechanism against [pazopanib + HDAC inhibitors] was activation of ERBB1, and the *in vitro* and *in vivo* the lethality of [pazopanib + entinostat] was enhanced by the multi-kinase inhibitor neratinib. In melanoma, we also demonstrated that pazopanib interacted with the HDAC inhibitors sodium valproate and AR42 to suppress tumor growth in athymic mice (3).

Axitinib is an approved multi-kinase inhibitor for renal carcinoma which blocks signaling from VEGFR family and PDGFR family receptor tyrosine kinases (4, 5). Other clinical data demonstrated that in patients who were refractory to axitinib the HDAC inhibitor vorinostat could be safely combined with the anti-VEGFR antibody bevacizumab (6). As a single agent, axitinib has a plasma C max of ~130 nM for a standard of care 5 mg tablet ingestion, with a relatively short ~6h half-life and has similar negative sequelae to drugs with an overlapping kinase inhibitory profile, e.g. planar hand-foot syndrome. Although the earliest research studies for axitinib date back to 2005, the majority of the literature discussing the drug are examining its *in vivo* and in-patient biology and efficacy. More significantly, the vast majority of axitinib laboratory-based studies have used the drug at one-two orders of magnitude too high a concentration. As of 2020, no truly agnostic mechanistic studies have been performed to determine whether axitinib combined with HDAC inhibitors would enhance its efficacy, or whether that or similar combinations could enhance the effectiveness of checkpoint immunotherapy antibodies.

In multiple prior studies combining multi-kinase inhibitors such as sorafenib and pazopanib with HDAC inhibitors, we noted that three important alterations in cell signaling occurred which were all vital for causing tumor cell death (7–10). The first was inhibition of receptors and signaling through the PI3K/AKT pathway. The second was the induction of a DNA damage/reactive oxygen species signal mediated by ATM through the AMPK and targeting both ULK1 and Raptor. The third was dysregulation of chaperone function resulting in endoplasmic reticulum stress signaling and eIF2α phosphorylation. The first two events led to inactivation of mTORC1 and mTORC2 and the activation of ULK1 followed by phosphorylation of the ULK1 substrate ATG13. ATG13 phosphorylation is the gatekeeping step required for autophagosome formation. Inactivation of eIF2α results in the expression of proteins with short half-lives declining, e.g. MCL-1, as well as increased expression of stress/autophagy related proteins such as Beclin1 and ATG5. Reduced levels of MCL-1 and BCL-XL also will cause the levels of ‘free’ Beclin1 in the cell to rise, thus further facilitating autophagy. Knock down of autophagy regulatory genes such as Beclin1, ATG5 and ATG16L1 or lysosomal proteases such as cathepsin B significantly reduced drug combination lethality. Similar findings were made over-expressing GRP78 or an activated form of mTOR or knocking down eIF2α expression.

The present manuscript set out to define the biology of axitinib alone and when combined with HDAC inhibitors. Furthermore, we would perform our work using clinically relevant concentrations of axitinib as well as of the HDAC inhibitors. Our findings provide compelling evidence that axitinib combines with HDAC inhibitors to kill sarcoma cells.

## Materials and Methods

### Materials

Entinostat, vorinostat, sodium valproate and axitinib were purchased from Selleck Chem (Houston, TX). Trypsin-EDTA, DMEM, RPMI, penicillin-streptomycin were purchased from GIBCOBRL (GIBCOBRL Life Technologies, Grand Island, NY). Sarcoma tumor cell lines were purchased from the ATCC and the NCI repository and were not further validated beyond that claimed by the vendors. HT1080 is a human fibrosarcoma isolate from a Caucasian male and expresses a mutant N-RAS; MES is a uterine sarcoma hypo-diploid isolate from a Caucasian. The mouse sarcoma tumors SAL and SAL/N are fibrosarcoma. TSC2 is a cutaneous sarcoma from a TSC2 +/- mouse. SAR 180 cells are a soft tissue sarcoma. Cells were re-purchased every ~6 months. All engineered plasmids were purchased from Addgene, (Cambridge, MA). Commercially available validated short hairpin RNA molecules to knock down RNA/protein levels were from Qiagen (Valencia, CA) (e.g. knock down data shown in **Supplemental Figures 1–6**). Antibodies to BAX, BAK, BCL-XL, CHOP, c-FLIP, IRE1, RIP1, iNOS, FADD, Cathepsin B, mTOR, phospho-mTOR S2448 and S2481, phospho-RAPTOR S722 & S792, TSC2 T1426, PTEN, phospho-PTEN S380, ATF6, eNOS, AIF, XBP1, NOXA, PUMA, ATG5 phospho- ATG13 S318, Beclin-1, AKT, phospho-AKT T308, eiF2α, phospho-eiF2α S51, phospho p65 S536, ATF4, PGKI and II, TRX, SOD, ATM, phospho-ATM S1981, AMPKα, phospho-AMPK T172, phospho-ULK1 S757, S317, STAT3, STAT5, p70 S6K, phospho-ERK1/2, GRP78, HSP70 and HSP90, phospho-γH2AX, were purchased from Cell Signaling Technology, (Danvers, MA). PERK, CD95 and caspase 9 antibodies, were purchased from Santa Cruz Biotechnology, (Dallas, TX) (1–3, 7–10).

### Methods

#### Culture and *In Vitro* Exposure of Cells to Drugs

All cell lines were cultured at 37°C (5% v/v CO_2_) *in vitro* using DMEM supplemented with 5% (v/v) fetal calf serum. *In vitro* drug treatments were generally from a 100 mM stock solution of each drug and the maximal concentration of Vehicle carrier (VEH; DMSO) in media was 0.02% (v/v). All drug stock solutions were stored at -80°C. Cells were not cultured in reduced serum media during any study in this manuscript.

#### Transfection of Cells With siRNA or With Plasmids

*For Plasmids:* Cells were transfected 24h after plating. Plasmids expressing a specific mRNA (or siRNA) or appropriate vector control plasmid DNA was diluted in 50 μl serum-free and antibiotic-free medium (1 portion for each sample). Concurrently, 2 μl Lipofectamine 2000 (Invitrogen), was diluted into 50 μl of serum-free and antibiotic-free medium (1 portion for each sample). Diluted DNA was added to the diluted Lipofectamine 2000 for each sample and incubated at room temperature for 30 min. This mixture was added to each well/dish of cells containing 200 μl serum-free and antibiotic-free medium for a total volume of 300 μl, and the cells were incubated for 4 h at 37°C. An equal volume of 2x medium was then added to each well. Cells were incubated for 24h, then treated with drugs.

*Transfection for siRNA:* Cells from a fresh culture growing in log phase as described above, were transfected 24h after plating. Prior to transfection, the medium was aspirated, and serum-free medium was added to each plate. For transfection, 10 nM of the annealed siRNA, the positive sense control doubled stranded siRNA targeting GAPDH or the negative control (a “scrambled” sequence with no significant homology to any known gene sequences from mouse, rat or human cell lines) were used. Ten nM siRNA (scrambled or experimental) was diluted in serum-free media. Four μl Hiperfect (Qiagen) was added to this mixture and the solution was mixed by pipetting up and down several times. This solution was incubated at room temp for 10 min, then added drop wise to each dish. The medium in each dish was swirled gently to mix, then incubated at 37°C for 2h. Serum-containing medium was added to each plate, and cells were incubated at 37°C for 24h before then treated with drugs (0-24h). Additional immuno-fluorescence/live-dead analyses were performed at the indicated time points. **Supplemental Figures S1–S6** show immuno-fluorescent imaging of a range of cell proteins following specific siRNA knockdown/or the use of a “scrambled” sequence with no significant homology to any known gene sequences from mouse, rat or human cell lines.

#### Assessments of Autophagosome and Autolysosome Levels

Cells were transfected with a plasmid to express LC3-GFP-RFP (Addgene #168997), and as indicated with siRNA molecules. Twenty-four h after transfection, cells are treated with vehicle control or the drugs, alone or in combination as indicated. Cells were imaged at 60X magnification 4 h and 8 h after drug exposure and the mean number of GFP+ and RFP+ punctae per cell determined from >50 randomly selected cells per condition. In the neutral autophagosome both GFP and RFP fluoresce whereas in the acidic autolysosome GFP is quenched and only RFP fluoresces. Thus, the appearance and disappearance of GFP+/RFP+ and RFP+ vesicles over time permits the detection of autophagosome formation and autophagic flux where autophagosomes fuse with acidic lysosomes to form autolysosomes. Studies were performed with three independent triplicates used to calculate the mean number of punctae per cell.

#### Animal Studies

Studies were performed according to USDA regulations under VCU IACUC protocol AD20008. Immuno-competent A/J mice (SAL cell line) or Swiss Webster (Sarcoma 180 cell line) (~20 g) were injected with 5 x 10^5^ cells into their rear flank (10 animals per treatment group for A/J and 8 per group Swiss Webster +/- SD). Tumors were permitted to form for 10 days with tumors at that time exhibiting a mean volume of ~50 mm^3^-~100 mm^3^, respectively. For studies with entinostat, mice were treated by oral gavage once every day QD for twenty-one days with vehicle (0.5% carboxymethyl cellulose, Sigma- Aldrich, St Louis MO, 63013, USA) or with axitinib (2 mg/kg). Animals received entinostat (1 mg/kg) on days 1, 4, 8, 12, 15, 18. For studies with valproate, mice were treated by oral gavage once every day QD for twenty days with vehicle (0.5% carboxymethyl cellulose, Sigma- Aldrich, St Louis MO, 63013, USA) or with [axitinib (2 mg/kg) plus sodium valproate (50 mg/kg)]. The body mass and volume of each tumor was assessed every 3-4 days using a digital caliper and tumor volume calculated using the equation volume = (L x W^2^)/2.

#### Detection of Cell Death by Trypan Blue

Trypan blue exclusion was used to assess cell viability at each experimental time point. Floating cells were isolated along with attached cells that were harvested by trypsinization with Trypsin/EDTA for ~3 min at 37°C. Following isolation, the total cell population for each experimental point was assessed for cell viability. Data are the means of three separate treatments, each from a triplicate determination (+/- SD).

#### Detection of Protein Expression and Protein Phosphorylation by In-Cell Western Blotting Using a Hermes WiScan Microscope

Cells (4 x 10^3^) were plated into 96 well plates and allowed to grow over night. Depending upon the specific experiment, cells were then either genetically manipulated, or treated with drugs. For genetic manipulation, cells were transfected with plasmids or siRNA molecules and incubated for an additional 24h. Cells were then treated with vehicle control or with drugs at the indicated final concentrations, alone or in combination. Cells were isolated/fixed for processing at various times following drug exposure. For immunofluorescence studies, after centrifugation, cell growth media was removed, and cells were fixed in place in 0.4% paraformaldehyde for 10 minutes at room temperature. The cells were then permeabilized using ice cold PBS containing 0.5% (v/v) Triton X-100. After 30 min the cells were washed three times with ice cold PBS and pre-blocked with rat serum for 3 hours. Following pre-blocking, cells were incubated with a primary antibody for the detection and expression/phosphorylation of a given protein (usually at 1:100 dilution from a commercial vendor) overnight at 37°C. Following overnight incubation, cells were washed three times with PBS followed by incubation with a secondary antibody containing an associated fluorescent red or green chemical tag, for 3 hours. Following this incubation, the cells were washed three times in PBS and 100 μl of PBS was added to each well for assessment of protein expression *via* microscopy. The cells were visualized at either 10X or 60X magnification in the Hermes wide-field microscope based on how data is to be presented. All immunofluorescent images for each individual protein/phospho-protein were recorded using the standardized microscope settings to ensure that signal level for each image was directly comparable to signal level in the control and drug treated cells. All phospho-protein values are corrected for the total expression of the protein, unless otherwise indicated. For other proteins, total loading controls used total invariant ERK2 expression. A minimum of 100 cells per treatment condition are randomly visualized by the microscope without any experimenter input, and the mean fluorescence intensity for those 100 cells is provided by the machine. This experiment is independently performed three times to provide a mean fluorescence value for a total of 300 cells/data points i.e. three mean values. Fluorescence intensity values for all treatment conditions are plotted and the mean percentage of fluorescence change compared to control, defined as 100%, is plotted (+/- SD).

#### Data Analysis

Comparison of the effects of various treatments was performed using one-way analysis of variance and a two tailed Student’s t-test using Sigma-Plot 13.0 and Sigma-Stat software corrected for multiple comparisons. Statistical examination of *in vivo* animal survival data utilized log rank statistical analyses between the different treatment groups. Differences with a p-value of < 0.05 were considered statistically significant. All experiments presented are the means of multiple individual points from multiple experiments (+/- SD).

## Results

**Figure 1:**
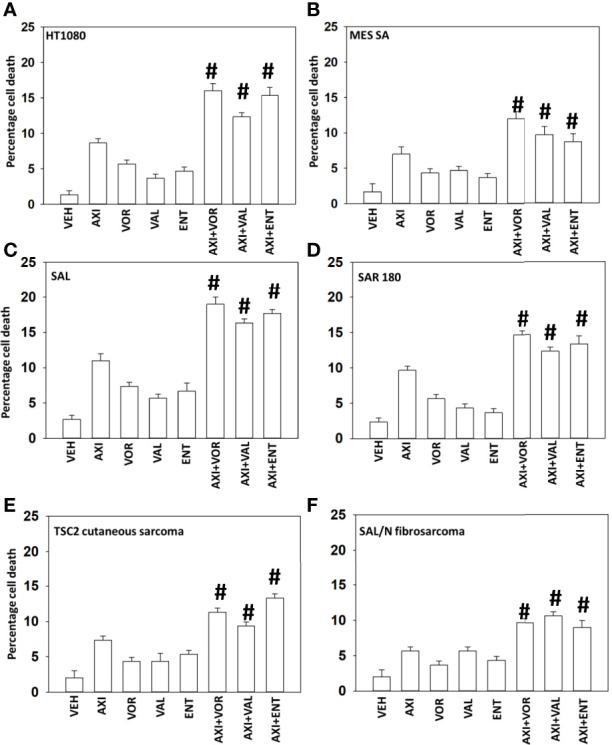
Axitinib interacts with HDAC inhibitors to kill sarcoma cells. **(A–F)** Sarcoma cells human (HT1080, MES) and mouse (SAL, SAR180, TSC2, SAL/N) were treated with vehicle control, axitinib (50 nM), entinostat (50 nM), vorinostat (500 nM), sodium valproate (250 μM) alone or in combination as indicated in the graphical panels. Twenty-four h later, cells were isolated, and viability determined by trypan exclusion assays in triplicate (n = 3 +/-SD). ^#^p < 0.05 greater than axitinib alone value.

Initial studies defined the ability of axitinib to interact with HDAC inhibitors to kill sarcoma cells. Using clinically achievable drug concentrations, in multiple human and rodent sarcoma cells, axitinib and HDAC inhibitors interacted in an additive to greater than additive fashion to kill (**Figures 1A–F**).

**Figure 2:**
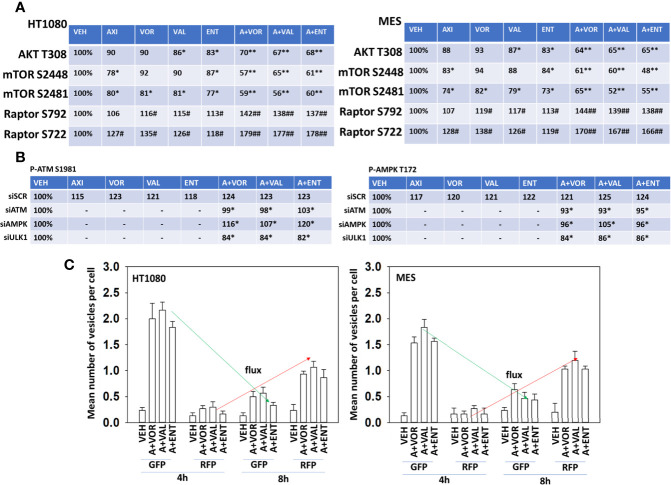
Axitinib combined with HDAC inhibitors inactivates AKT, mTORC1, mTOR2 and Raptor. **(A)** Human sarcoma cells were treated with vehicle control, axitinib (50 nM), entinostat (50 nM), vorinostat (500 nM), sodium valproate (250 μM) alone or in combination as indicated in the graphical panels for 6h. Cells were fixed in place and in-cell immunoblotting performed to determine the total protein levels of AKT, mTOR and Raptor, and the phosphorylation of AKT T308, mTOR S2448, mTOR S2481, Raptor S792 and Raptor S722. The percentage phosphorylation is stated for each site corrected for total protein loading with vehicle control defined as 100% (n = 3 +/-SD). *p < 0.05 less than vehicle control; **p < 0.05 less than HDAC inhibitor alone; ^#^p < 0.05 greater than vehicle control; ^##^p < 0.05 greater than HDAC inhibitor alone. **(B)** HT1080 cells were transfected with a scrambled control siRNA (siSCR) or with validated siRNA molecules to knock down the expression of either ATM or AMPKα. Twenty-four h after transfection, cells were treated with vehicle control, axitinib (50 nM), entinostat (50 nM), vorinostat (500 nM), sodium valproate (250 μM) alone or in combination as indicated in the graphical panels for 6h. Cells were fixed in place and in-cell immunoblotting performed to determine the total protein levels of ATM and AMPKα, and the phosphorylation of ATM S1981 and AMPKα T172 determined. The percentage phosphorylation is stated for each site corrected for total protein loading with vehicle control of each transfection being defined as 100% (n = 3 +/-SD). ^#^p < 0.05 greater than vehicle control value; *p < 0.05 less than corresponding value in siSCR cells. **(C)** Sarcoma cells were transfected with a plasmid to express LC3-GFP-RFP. Twenty-four h after transfection, cells were treated with vehicle control, [axitinib (50 nM) + entinostat (50 nM)], [axitinib (50 nM) + vorinostat (500 nM)], [axitinib (50 nM) + sodium valproate (250 μM)] in combination as indicated in the graphical panels for 4h and 8h. The mean number of intense green and red punctae from a minimum of 40 cells are defined and plotted (n = 3 +/-SD).

Multiple prior studies from our laboratory have demonstrated that HDAC inhibitors can cause a DNA damage response which results in autophagosome formation. Hence, we next performed mechanism-based screening studies to assess the impact of axitinib and HDAC inhibitor exposure on alterations in signal transduction pathway activities. Axitinib and HDAC inhibitors interacted in a greater than additive fashion to reduce the phosphorylation of AKT T308, mTOR S2448, mTOR S2481 and to enhance in a greater than additive fashion the phosphorylation of Raptor S722 and S792 (**Figure 2A**). This would be predicted to enhance autophagosome formation.

Knock down of ATM prevented the drug combinations from increasing residual specific ATM S1981 phosphorylation (**Figure 2B**, left). Over-expression of TRX (thioredoxin) or SOD2 (superoxide dismutase 2) significantly reduced the ability of the drug combination to activate ATM (not shown). Knock down of AMPKα slightly reduced the ability of the drug combinations increasing ATM S1981 phosphorylation whereas knock down of ULK1 unexpectedly reduced basal and drug-stimulated ATM S1981 phosphorylation. Knock down of ATM significantly reduced the abilities of the drug combinations to enhance AMPKαT172 phosphorylation (**Figure 2B**, right). Knock down of AMPKα prevented the drug combinations from increasing residual AMPKα T172 phosphorylation. Again, knock down of ULK1 reduced basal AMPKα T172 phosphorylation and prevented the drug combinations from enhancing T172 phosphorylation. As would be predicted, based on activation of the AMPK and inactivation of mTOR, the drug combinations enhanced autophagosome formation that was followed later by the formation of autolysosomes (**Figure 2C**).

**Figure 3:**
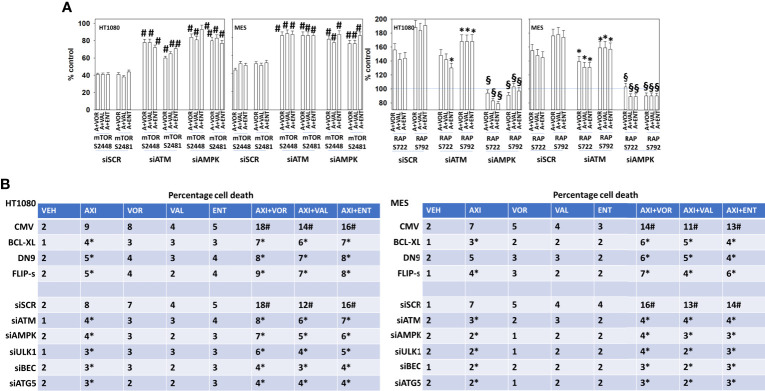
Signaling by ATM-AMPK plays a key role in regulating both mTOR and Raptor function. **(A)** HT1080 and MES sarcoma cells were transfected with a scrambled control siRNA (siSCR) or with validated siRNA molecules to knock down the expression of either ATM or AMPKα. Twenty-four h after transfection, cells were treated with vehicle control, [axitinib (50 nM) + entinostat (50 nM)], [axitinib (50 nM) + vorinostat (500 nM)], [axitinib (50 nM) + sodium valproate (250 μM)] in combination as indicated in the graphical panels for 6h. Cells were fixed in place and in-cell immunoblotting performed to determine the total protein levels of mTOR and Raptor, and the phosphorylation of mTOR S2448, mTOR S2481, Raptor S722 and Raptor S792. The percentage phosphorylation is plotted for each site corrected for total protein loading with vehicle control of each transfection being defined as 100% (n = 3 +/-SD). ^#^p < 0.05 greater than corresponding value in siSCR control; *p < 0.05 less than corresponding value in siSCR cells; ^§^p < 0.05 less than corresponding value in siATM cells. **(B)** Sarcoma cells were either transfected with an empty vector plasmid (CMV) or with plasmids to express BCL-XL, c-FLIP-s or dominant negative caspase 9; or were transfected with a scrambled siRNA control or with validated siRNA molecules to knock down the expression of ATM, AMPKα, ULK1, ATG5 and Beclin1. Twenty-four h after transfection, cells were treated with vehicle control, axitinib (50 nM), entinostat (50 nM), vorinostat (500 nM), sodium valproate (250 μM) alone or in combination as indicated in the graphical panels. Twenty-four h later, cells were isolated, and viability determined by trypan exclusion assays in triplicate (n = 3 +/-SD). Data are rounded to the nearest whole number. *p < 0.05 less than corresponding value in siSCR/CMV transfected cells.

We next determined whether the phosphorylation of mTOR S2448, mTOR S2481, Raptor S722 and S792 were mediated by ATM-AMPK signaling. Knock down of either ATM or of AMPKα significantly reduced any of our axitinib-based drug combinations from decreasing mTOR S2448/S2481 phosphorylation or from increasing Raptor S722/S792 phosphorylation (**Figure 3A**). Over-expression of BCL-XL, FLIP-s or dominant negative caspase 9 significantly reduced the lethality of all drug combinations (**Figure 3B**). Knock down of ATM, AMPKα, ULK1, Beclin1 or ATG5 also protected sarcoma cells from drug combination-induced killing, with knock down of Beclin1 or ATG5 apparently more protective than over-expression of BCL-XL, FLIP-s and dominant negative caspase 9.

**Figure 4:**
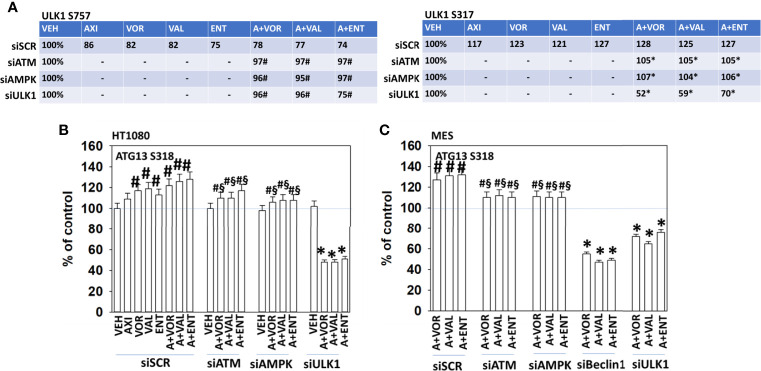
Alterations in ULK1 phosphorylation require ATM-AMPK signaling; autophagy is required for cell killing. **(A)** HT1080 cells were transfected with a scrambled control siRNA (siSCR) or with validated siRNA molecules to knock down the expression of either ATM or AMPKα. Twenty-four h after transfection, cells were treated with vehicle control, axitinib (50 nM), entinostat (50 nM), vorinostat (500 nM), sodium valproate (250 μM) alone or in combination as indicated in the graphical panels for 6h. Cells were fixed in place and in-cell immunoblotting performed to determine the total protein levels of ULK1 and the phosphorylation of ULK1 S317 and ULK1 S757. The percentage phosphorylation is stated for each site corrected for total protein loading with vehicle control of each transfection being defined as 100% (n = 3 +/-SD). ^#^p < 0.05 greater than vehicle control; *p < 0.05 less than vehicle control. **(B, C)** HT1080 and MES cells were transfected with a scrambled control siRNA (siSCR) or with validated siRNA molecules to knock down the expression of either ATM or AMPKα. Twenty-four h after transfection, cells were treated with vehicle control, axitinib (50 nM), entinostat (50 nM), vorinostat (500 nM), sodium valproate (250 μM) alone or in combination as indicated in the graphical panels for 6h. Cells were fixed in place and in-cell immunoblotting performed to determine the total protein levels of ATG13 and the phosphorylation of ATG13 S318. The percentage phosphorylation is plotted for ATG13 S318, corrected for total ATG13 protein loading with vehicle control of each transfection being defined as 100% (n = 3 +/-SD). ^#^p < 0.05 greater than vehicle control; ^§^p < 0.05 less than corresponding value in siSCR cells; *p < 0.05 less than vehicle control.

Further molecular analyses of interlinked signaling pathways were performed. In cells exposed to axitinib and HDAC inhibitors, the phosphorylation of ULK1 S757 declined and the phosphorylation of ULK1 S317 was enhanced (**Figure 4A** and **Supplemental Figure 7**). Knock down of ATM or AMPKα prevented the drug-induced changes in ULK1 phosphorylation. The phosphorylation of ATG13 S318 is the key gate-keeping step that initiates autophagosome formation. Knock down of ATM or AMPKα partially, though significantly, reduced the drug combination-stimulated phosphorylation of ATG13 S318 (**Figures 4B, C**). Knock down of ULK1 did not alter basal ATG13 S318 phosphorylation but, unexpectedly, exposure of cells to the drug combinations in the absence of ULK1 caused a significant reduction in phospho-S318 levels. These data argue that our drug combinations activate an ATM-AMPK-ULK1 kinase signaling pathway but simultaneously also probably activate a protein phosphatase which can act to rapidly inactivate ULK1 and reduce ATG13 phosphorylation.

**Figure 5:**
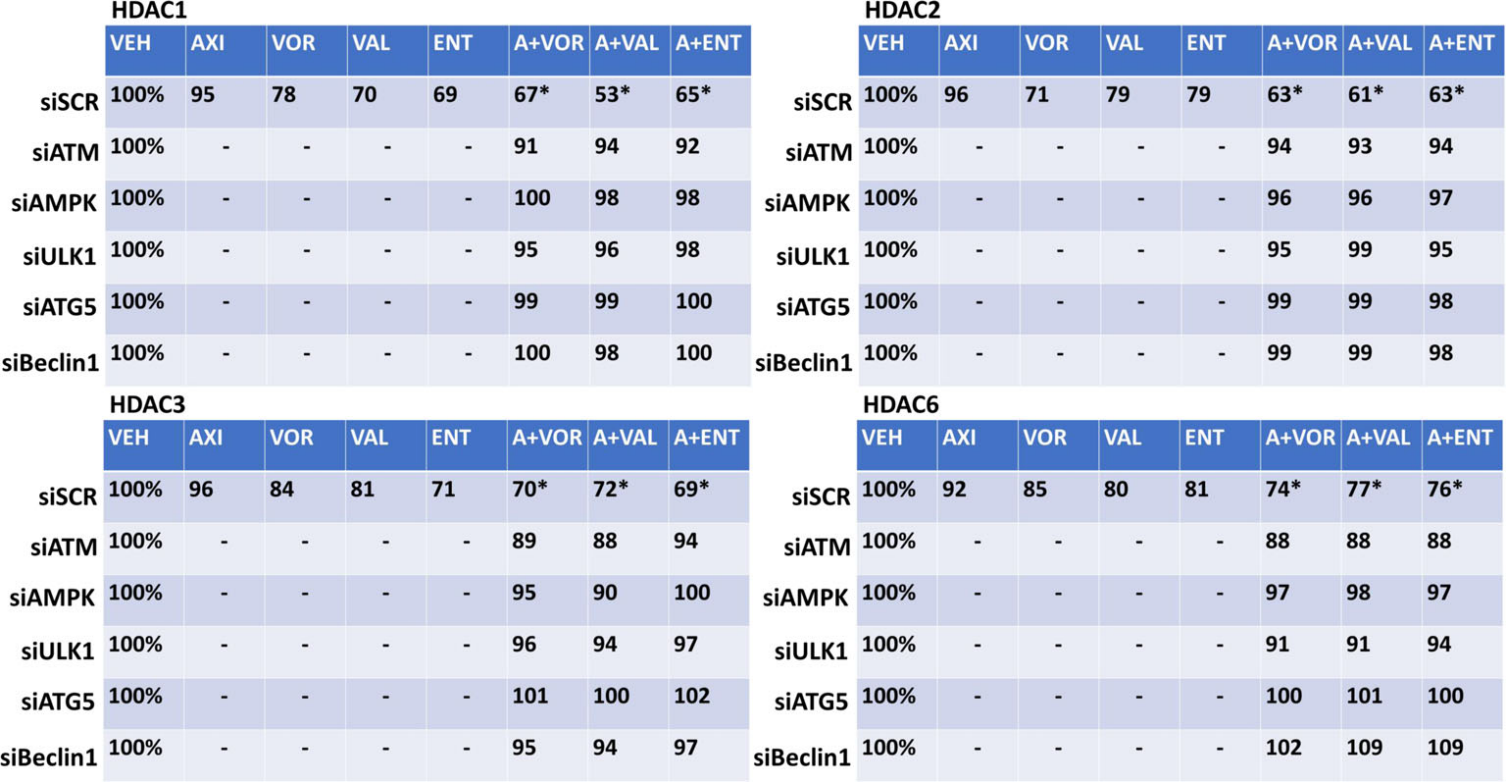
Axitinib combined with HDAC inhibitors reduces the expression of multiple HDAC proteins in HT1080 cells *via* ATM-AMPK signaling and ULK1-autophagy. HT1080 cells were transfected with a scrambled control siRNA (siSCR) or with validated siRNA molecules to knock down the expression of ATM, AMPKα, ULK1, ATG5 or Beclin1. Twenty-four h after transfection, cells were treated with vehicle control, axitinib (50 nM), entinostat (50 nM), vorinostat (500 nM), sodium valproate (250 μM) alone or in combination as indicated in the graphical panels for 6h. Cells were fixed in place and in-cell immunoblotting performed to determine the total protein levels of HDACs 1, 2, 3, and 6, and of invariant ERK2. The percentage expression is presented for each HDAC protein corrected for total ERK2 protein loading with vehicle control of each transfection being defined as 100% (n = 3 +/-SD). *p < 0.05 less than HDAC inhibitor alone exposure.

The expression of HDAC proteins can be rapidly reduced *via* autophagy. In HT1080 and MES cells, axitinib and HDAC inhibitors interacted to reduce and further reduce the expression of multiple HDAC proteins including HDACs 1, 2, 3, 4, 5 and 6 (**Figure 5** and **Supplemental Figures 8**, **9**). Knock down of ATM, AMPKα, ULK1, ATG5 or Beclin1 significantly reduced the ability of the drug exposures to lower HDAC protein levels. This strongly suggests, as observed in prior studies, that our drug combination, *via* ATM-AMPK-ULK1 signaling and enhanced autophagosome formation, reduces HDAC protein expression. Treatment of HT1080 and MES sarcoma cells with axitinib and HDAC inhibitors reduced the expression of multiple HDAC proteins, an effect that was blocked by expression of an activated form of mTOR, previously shown by ourselves and others to prevent autophagosome formation (**Supplemental Figures 10**, **11**).

Altered expression levels of HDAC proteins will in turn control the transcriptome of the cell. In the case of proteins that regulate sensitivity to checkpoint immunotherapies, we have previously observed that drug combinations which induce autophagosome formation and that down-regulate HDAC expression also enhance expression of Class I MHCA and reduce the levels of PD-L1. In human sarcoma cells, exposure to axitinib and HDAC inhibitors rapidly increased the expression of MHCA and reduced the levels of PD-L1, PD-L2, ornithine decarboxylase (ODC) and indoleamine 2, 3-dioxygenase 1 (IDO1) (**Supplemental Figures 12A, B**). In mouse sarcoma cells, the drug combinations decreased PD-L1 levels and enhanced MHCA expression (**Supplemental Figures 13A–D**). The expression of ODC also trended to decline. Knock down of HDAC proteins was then performed to define which HDACs played key roles in controlling protein expression. Knock down of [HDAC1 + HDAC2] or [HDAC2 + HDAC3] significantly reduced the changes in protein expression (**Supplemental Figure 14**). Knock down of [HDAC1 + HDAC3] almost eliminated any changes in protein expression caused by the drug combinations. Thus, the axitinib plus HDAC inhibitor combinations inactivate mTOR and enhance autophagosome formation. The expression of HDAC proteins, *via* autophagic processes, are reduced, which alters gene transcription, resulting in a putatively enhanced immunogenic profile.

**Figure 6:**
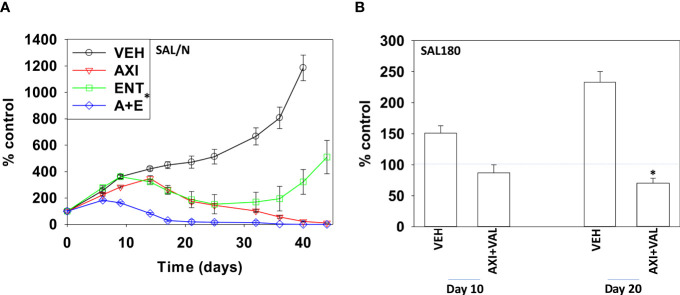
Axitinib and HDAC inhibitors interact to suppress the growth of mouse sarcoma tumors grown in their syngeneic host. **(A)** SAL/N tumors were treated with vehicle control, axitinib, entinostat or the drug combination for 21 days as described in the Methods. Tumor volumes were measured for 44 days after the initiation of treatment. (n = 10 animals per group +/-SD) *p < 0.05 less than axitinib alone growth. **(B)** SAL180 tumors were grown to a volume of ~100 mm^3^ and then treated with either vehicle control or with [axitinib + valproate] for 20 days as described in the Methods. Tumor volumes were measured (n = 8 animal per group +/-SD). *p < 0.05 less than vehicle control.

Finally, we performed studies *in vivo* to determine whether axitinib interacted with HDAC inhibitors to suppress the growth of sarcoma tumors. SAL/N tumors were formed in their syngeneic host mouse. Mice were treated for 21 days with vehicle control, axitinib, entinostat or the drugs combined. Tumors in animals treated with entinostat exhibited growth arrest flowed by tumor regrowth (**Figure 6A**). Drug exposure did not alter animal body mass (not shown). Tumors treated with axitinib also grew during therapy only for tumor growth to cease, flowed by a slow decline in tumor volume until by day 44 tumors were undetectable. In mice treated with the drug combination, tumor growth was more rapidly suppressed, and the combination caused a significantly more rapid reduction in tumor mass compared to axitinib alone. Sarcoma 180 tumors were established in Swiss Webster mice and treated with vehicle control or with [axitinib + valproate] for 20 days. As was observed in the SAL/N tumors, the drug combination significantly reduced Sarcoma 180 tumor growth (30% at Day 20). Again, no change in animal body mass was observed (**Figure 6B**; not shown).

## Discussion

**Figure 7:**
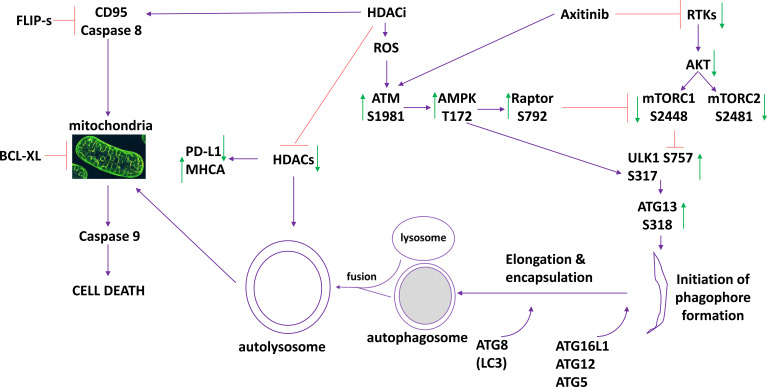
The molecular mechanisms by which axitinib and HDAC inhibitors interact to kill sarcoma cells. Axitinib inactivates RTKs and downstream AKT/mTOR signaling is reduced. Axitinib and HDAC inhibitors both activate ATM. ATM activation results in activation of the AMPK and Raptor. This results in a greater inactivation of mTOR and complete activation of ULK1. ULK1 phosphorylates ATG13 which promotes autophagosome formation followed by autophagic flux and the formation of autolysosomes. HDAC proteins are inhibited by HDACi but these proteins are also digested in the autolysosomes reducing their total protein levels. This directly impacts upon the expression of PD-L1 (reduced) and MHCA (enhanced). HDACs can increase the expression and trimerization of the death receptor CD95. Inhibition of caspase 8/10 by FLIP-s significantly reduced cell killing. Autolysosome proteases such as cathepsins can cause mitochondrial dysfunction, as can caspase 8 signaling. Over-expression of the mitochondrial protective protein BCL-XL significantly reduced killing. Downstream of the mitochondrion, killing can be mediated by cytochrome c/caspase 9/caspases 3 and 7 or by AIF. Our data, by expressing dominant negative caspase 9, argues that the majority of the death signal is mediated *via* caspase 9/caspase 3.

The present studies were performed to define the molecular mechanisms by which low clinically achievable concentrations of axitinib interact with HDAC inhibitors to kill sarcoma cells. The drug combinations activated an ATM-AMPK pathway which was responsible for phosphorylation of Raptor, inactivation of mTORC1 and mTORC2, activation of ULK1 and elevated ATG13 S318 phosphorylation. Cell killing was reduced by expression of FLIP-s, BCL-XL or dominant negative caspase 9, and was abolished by knock down of Beclin1 or ATG5 (**Figure 7**). Collectively this data argues for the drug combination causing a primary activation of ATM-AMPK signaling which leads to autophagosome formation that in turn is followed by mitochondrial dysfunction and killing through apoptotic and non-apoptotic mechanisms.

We have previously published that autophagosome formation and autophagic flux can reduce the expression of multiple HDAC proteins (11, 12). As single agents, axitinib, entinostat, vorinostat and valproate all exhibited to some degree an ability to reduce HDAC levels, however generally, the combination of axitinib with an HDAC inhibitor resulted in a significantly greater reduction in HDAC expression. Notably, the drug combinations all lowered the levels of HDAC1, HDAC2 and HDAC3 by almost 30% within 6h. The drug-induced reduction of HDAC levels was blocked by knockdown of autophagosome regulatory proteins and by expression of a mutant active form of mTOR. In the absence of [HDAC1 + HDAC2], [HDAC1 + HDAC3] or [HDAC2 + HDAC3] the [axitinib + HDAC inhibitor] combinations could not increase expression of MHCA nor lower expression of PD-L1. And, the ability of our drug combinations to enhance ATG13 S318 phosphorylation required ATM-AMPK signaling. These data collectively demonstrate that drug combinations, by causing ATM-AMPK-autophagy lead to the degradation of HDAC proteins, hence regulating transcription *via* autophagy.

At present, Medline lists 15 manuscripts when searching for axitinib and sarcoma, none of which combine axitinib with HDAC inhibitors. Several clinical trials have combined axitinib with checkpoint inhibitory immunotherapeutic antibodies (13–15). Axitinib plus pembrolizumab caused significant (7/21) but manageable toxicity and the combination displayed activity in patients with advanced sarcomas (15). As a single agent, axitinib has displayed anti-tumor activity in several sarcoma subtypes (16, 17). This included findings that patients who had progressed on pazopanib had subsequently responded to axitinib. Our data demonstrated that axitinib and HDAC inhibitors interacted *in vitro* and *in vivo* to kill tumor cells and prolong animal survival. This is similar to our prior findings combining pazopanib with HDAC inhibitors (1, 2).

In conclusion, our data has shown that axitinib and HDAC inhibitors interact *in vitro* and *in vivo* to kill tumor cells. Activation of an ATM-AMPK-autophagy pathway resulting in mitochondrial dysfunction plays an essential role in the killing process. Based on the drug combination reducing PD-L1 and increasing MHCA levels, future studies will determine whether axitinib plus valproate enhances the efficacy of checkpoint inhibitory immunotherapy antibodies.

## Data Availability Statement

The original contributions presented in the study are included in the article/**Supplementary Material**. Further inquiries can be directed to the corresponding author.

## Ethics Statement

The animal study was reviewed and approved by Virginia Commonwealth University IACUC.

## Author Contributions

AP and PD conceived the studies. PD supervised the studies and wrote the manuscript. AP edited the manuscript. LB and JR performed the experimental studies. All authors contributed to the article and approved the submitted version.

## Funding

Support for the present study was funded from philanthropic funding from Massey Cancer Center and the Universal Inc. Chair in Signal Transduction Research. PD acknowledges funding by the Commonwealth Health Research Board (CHRB) of Virginia.

## Conflict of Interest

The authors declare that the research was conducted in the absence of any commercial or financial relationships that could be construed as a potential conflict of interest.

## Publisher’s Note

All claims expressed in this article are solely those of the authors and do not necessarily represent those of their affiliated organizations, or those of the publisher, the editors and the reviewers. Any product that may be evaluated in this article, or claim that may be made by its manufacturer, is not guaranteed or endorsed by the publisher.

## Abbreviations

ERK, extracellular regulated kinase; PI3K, phosphatidyl inositol 3 kinase; ca, constitutively active; dn, dominant negative; ER, endoplasmic reticulum; AIF, apoptosis inducing factor; AMPK, AMP-dependent protein kinase; mTOR, mammalian target of rapamycin; JAK, Janus Kinase; STAT, Signal Transducers and Activators of Transcription; MAPK, mitogen activated protein kinase; PTEN, phosphatase and tensin homologue on chromosome ten; ROS, reactive oxygen species; CMV, empty vector plasmid or virus; si, small interfering; SCR, scrambled; PDE, phospho-diesterase; IP, immunoprecipitation; VEH, vehicle; AXI, axitinib; VAL, valproate; VOR, vorinostat; ENT, entinostat; HDAC, histone deacetylase; PD-L1, programed death ligand 1; ODC, ornithine decarboxylase; IDO1, Indoleamine 2,3-dioxygenase; MHCA, Human Major Histocompatibility Complex Class I A; RAP, Raptor.
