# Supplementary material for: Axitinib and HDAC Inhibitors Interact to Kill Sarcoma Cells
Source: Front Oncol. 2021 Sep 16;11:723966. doi: 10.3389/fonc.2021.723966 (PMC8483767; doi:10.3389/fonc.2021.723966)

Supplemental Figure 1. Control data showing siRNA knock down in HT1080 cells. Cells were transfected with siRNA molecules as described in the Methods. Twenty-four h after transfection, cells were fixed in place and in cell immunostaining performed to determine the expression of each protein and total ERK2 expression as a loading control. Images at 60X magnification, numeric knock down values are corrected for total ERK2 levels under each condition.

Supplemental Figure 2. Control data showing protein over-expression in HT1080 cells. Cells were transfected with plasmids as described in the Methods. Twenty-four h after transfection, cells were fixed in place and in cell immunostaining performed to determine the expression of each protein and total ERK2 expression as a loading control. Images at 60X magnification, numeric over-expression values are corrected for total ERK2 levels under each condition.

Supplemental Figure 3. Control data showing siRNA knock down or protein over-expression in MES cells. Cells were transfected with siRNA molecules or plasmids as described in the Methods. Twenty-four h after transfection, cells were fixed in place and in cell immunostaining performed to determine the expression of each protein and total ERK2 expression as a loading control. Images at 60X magnification, numeric knock down and over expression values are corrected for total ERK2 levels under each condition.

Supplemental Figure 4. Validation data showing siRNA knock down of K-RAS and N-RAS in HT1080 cells and MES cells. Cells were transfected as described in the Methods. Twenty-four h after transfection, cells were fixed in place and in cell immunostaining performed to determine the expression of each protein and total ERK2 expression as a loading control. Representative images are presented at 60X magnification with invariant ERK2 as a loading control.

Supplemental Figure 5. Validation of anti-HDAC antibodies in HT1080 cells. Cells were transfected with siRNA molecules as described in the Methods. Twenty-four h after transfection, cells were fixed in place and in cell immunostaining performed to determine the expression of each protein and total ERK2 expression as a loading control. Images at 60X magnification, numeric knock down values are corrected for total ERK2 levels under each condition.

Supplemental Figure 6. Validation of anti-HDAC antibodies in MES cells. Cells were transfected with siRNA molecules as described in the Methods. Twenty-four h after transfection, cells were fixed in place and in cell immunostaining performed to determine the expression of each protein and total ERK2 expression as a loading control. Images at 60X magnification, numeric knock down values are corrected for total ERK2 levels under each condition.

Supplemental Figure 7. Alterations in ULK1 phosphorylation require ATM-AMPK signaling; autophagy is required for cell killing. MES cells were transfected with a scrambled control siRNA (siSCR) or with validated siRNA molecules to knock down the expression of either ATM or AMPKα. Twenty-four h after transfection, cells were treated with vehicle control, axitinib (50 nM), entinostat (50 nM), vorinostat (500 nM), sodium valproate (250 μM) alone or in combination as indicated in the graphical panels for 6h. Cells were fixed in place and in-cell immunoblotting performed to determine the total protein levels of ULK1 and the phosphorylation of ULK1 S317 and ULK1 S757. The percentage phosphorylation is plotted for each site corrected for total protein loading with vehicle control of each transfection being defined as 100% (n = 3 +/-SD). # $p < 0.05$ greater than vehicle control; * $p < 0.05$ less than vehicle control; ¶ $p < 0.05$ greater than corresponding vehicle control value in siSCR cells; ∞ $p < 0.05$ greater than corresponding value in siSCR cells; † $p < 0.05$ less than corresponding value in siSCR cells.

Supplemental Figure 8. Axitinib combined with HDAC inhibitors reduces the expression of multiple HDAC proteins in HT1080 cells via ATM-AMPK signaling and ULK1-autophagy. HT1080 cells were transfected with a scrambled control siRNA (siSCR) or with validated siRNA molecules to knock down the expression of ATM, AMPKα, ULK1, ATG5 or Beclin1. Twenty-four h after transfection, cells were treated with vehicle control, axitinib (50 nM), entinostat (50 nM), vorinostat (500 nM), sodium valproate (250 μM) alone or in combination as indicated in the graphical panels for 6h. Cells were fixed in place and in-cell immunoblotting performed to determine the total protein levels of HDACs1-11 and of invariant ERK2. The percentage expression is plotted for each HDAC protein corrected for total ERK2 protein loading with vehicle control of each transfection being defined as 100% (n = 3 +/-SD). # $p < 0.05$ greater than corresponding values in siSCR control; * $p < 0.05$ less than HDAC inhibitor alone exposure.

Supplemental Figure 9. Axitinib combined with HDAC inhibitors reduces the expression of multiple HDAC proteins in MES cells via ATM-AMPK signaling and ULK1-autophagy. MES cells were transfected with a scrambled control siRNA (siSCR) or with validated siRNA molecules to knock down the expression of ATM, AMPKα, ULK1, ATG5 or Beclin1. Twenty-four h after transfection, cells were treated with vehicle control, axitinib (50 nM), entinostat (50 nM), vorinostat (500 nM), sodium valproate (250 μM) alone or in combination as indicated in the graphical panels for 6h. Cells were fixed in place and in-cell immunoblotting performed to determine the total protein levels of HDACs1-11 and of invariant ERK2. The percentage expression is plotted for each HDAC protein corrected for total ERK2 protein loading with vehicle control of each transfection being defined as 100% (n = 3 +/- SD). # $p < 0.05$ greater than corresponding values in siSCR control; * $p < 0.05$ less than HDAC inhibitor alone exposure.

Supplemental Figure 10. Expression of a mutant activated form of mTOR prevents the drug-induced degradation of HDAC proteins. HT1080 cells were transfected with an empty vector plasmid (CMV) or a plasmid to express a mutant activated form of mTOR. Twenty-four h after transfection, cells were treated with vehicle control, [axitinib (50 nM) + entinostat (50 nM)], [axitinib (50 nM) + vorinostat (500 nM)], [axitinib (50 nM) + sodium valproate (250 μM)] in combination as indicated in the graphical panels for 6h. Cells were fixed in place and in-cell immunoblotting performed to determine the total protein levels of HDACs1-11 and ERK2. The percentage expression is plotted for each HDAC protein corrected for total ERK2 protein loading with vehicle control of each transfection being defined as 100% (n = 3 +/-SD). # $p < 0.05$ greater than corresponding values in CMV control transfected.

Supplemental Figure 11. Expression of a mutant activated form of mTOR prevents the drug-induced degradation of HDAC proteins. MES cells were transfected with an empty vector plasmid (CMV) or a plasmid to express a mutant activated form of mTOR. Twenty-four h after transfection, cells were treated with vehicle control, [axitinib (50 nM) + entinostat (50 nM)], [axitinib (50 nM) + vorinostat (500 nM)], [axitinib (50 nM) + sodium valproate (250 μM)] in combination as indicated in the graphical panels for 6h. Cells were fixed in place and in-cell immunoblotting performed to determine the total protein levels of HDACs1-11 and ERK2. The percentage expression is plotted for each HDAC protein corrected for total ERK2 protein loading with vehicle control of each transfection being defined as 100% (n = 3 +/-SD). # $p < 0.05$ greater than corresponding values in CMV control transfected.

Supplemental Figure 12. Exposure of human sarcoma cells to [axitinib + HDAC inhibitor] reduces the expression of the immunotherapy biomarkers PD-L1, PD-L2, ODC and IDO1, and increases Class I MHCA levels. A and B. Human sarcoma cells (HT1080, MES) cells were treated with vehicle control, axitinib (50 nM), entinostat (50 nM), vorinostat (500 nM), sodium valproate (250 μM) alone or in combination as indicated in the graphical panels for 6h. Cells were fixed in place and in-cell immunoblotting performed to determine the total protein levels of PD-L1, PD-L2, MHCA, ODC and IDO1 and of invariant total ERK2. The percentage expression is plotted for each protein corrected for total ERK2 protein loading with vehicle control of each transfection being defined as 100% (n = 3 +/-SD). # $p < 0.05$ greater than vehicle control; * $p < 0.05$ less than vehicle control.

Supplemental Figure 13. Exposure of rodent sarcoma cells to [axitinib + HDAC inhibitor] reduces the expression of the immunotherapy biomarkers PD-L1, PD-L2, ODC and IDO1, and increases Class I MHCA levels. A.-D. Rodent sarcoma cells (SAL, SAR 180, TSC2, SAL/N) cells were treated with vehicle control, axitinib (50 nM), entinostat (50 nM), vorinostat (500 nM), sodium valproate (250 μM) alone or in combination as indicated in the graphical panels for 6h. Cells were fixed in place and in-cell immunoblotting performed to determine the total protein levels of PD-L1, PD-L2, MHCA, ODC and IDO1 and of invariant total ERK2. The percentage expression is plotted for each protein corrected for total ERK2 protein loading with vehicle control of each transfection being defined as 100% (n = 3 +/-SD). # $p < 0.05$ greater than vehicle control; * $p < 0.05$ less than vehicle control.

Supplemental Figure 14. Knock down of HDACs 1, 2, 3 recapitulates the effects on immunotherapy biomarkers of [axitinib + HDAC inhibitors]. HT1080 and MES cells were transfected with a scrambled control siRNA (siSCR) or with validated siRNA molecules to knock down the expression of HDAC1, HDAC2 or HDAC3; see graphical panels for the combinations of siRNAs used in each knock-down. Twenty-four h afterwards, cells were treated with vehicle control, [axitinib (50 nM) + entinostat (50 nM)], [axitinib (50 nM) + vorinostat (500 nM)], [axitinib (50 nM) + sodium valproate (250 μM)] in combination as indicated in the graphical panels for 6h. Cells were fixed in place and in-cell immunoblotting performed to determine the total protein levels of PD-L1, PD-L2, MHCA, ODC, IDO1 and invariant ERK2. The percentage expression is plotted for each protein corrected for total ERK2 protein loading with vehicle control of each transfection being defined as 100% (n = 3 +/-SD). # $p < 0.05$ greater than vehicle control; * $p < 0.05$ less than vehicle control.

Supplemental Figure 1

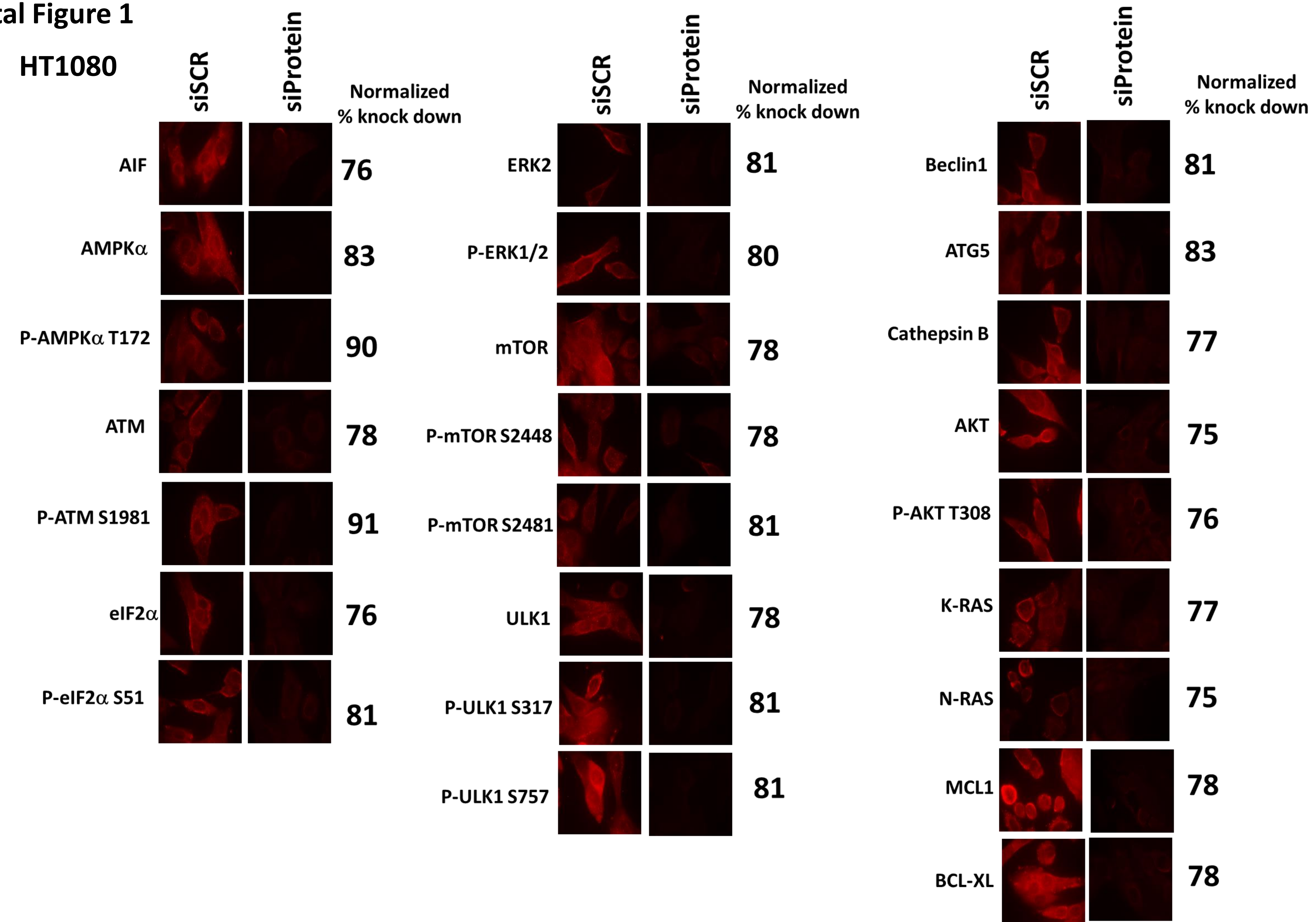

Supplemental Figure 2

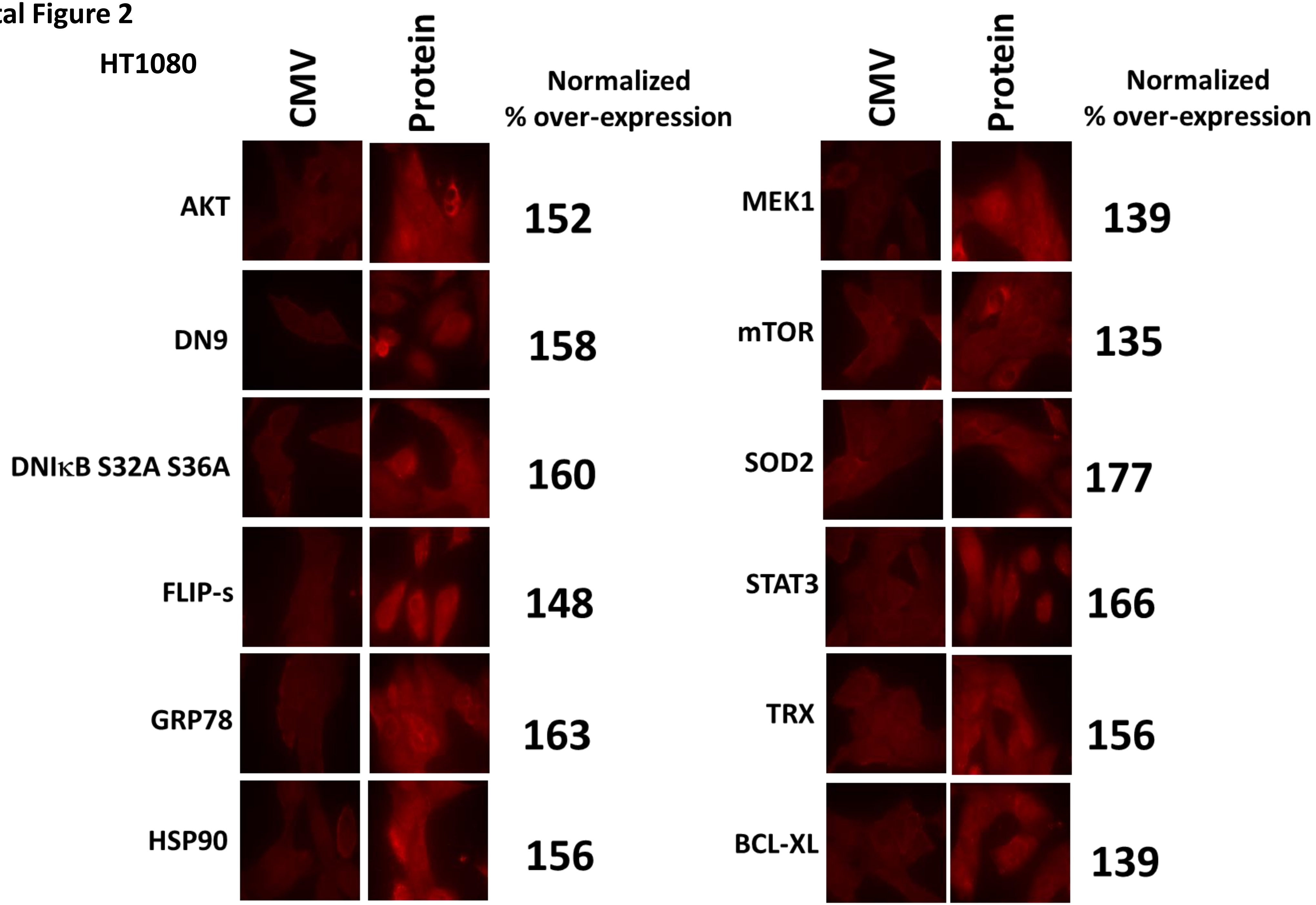

Supplemental Figure 3

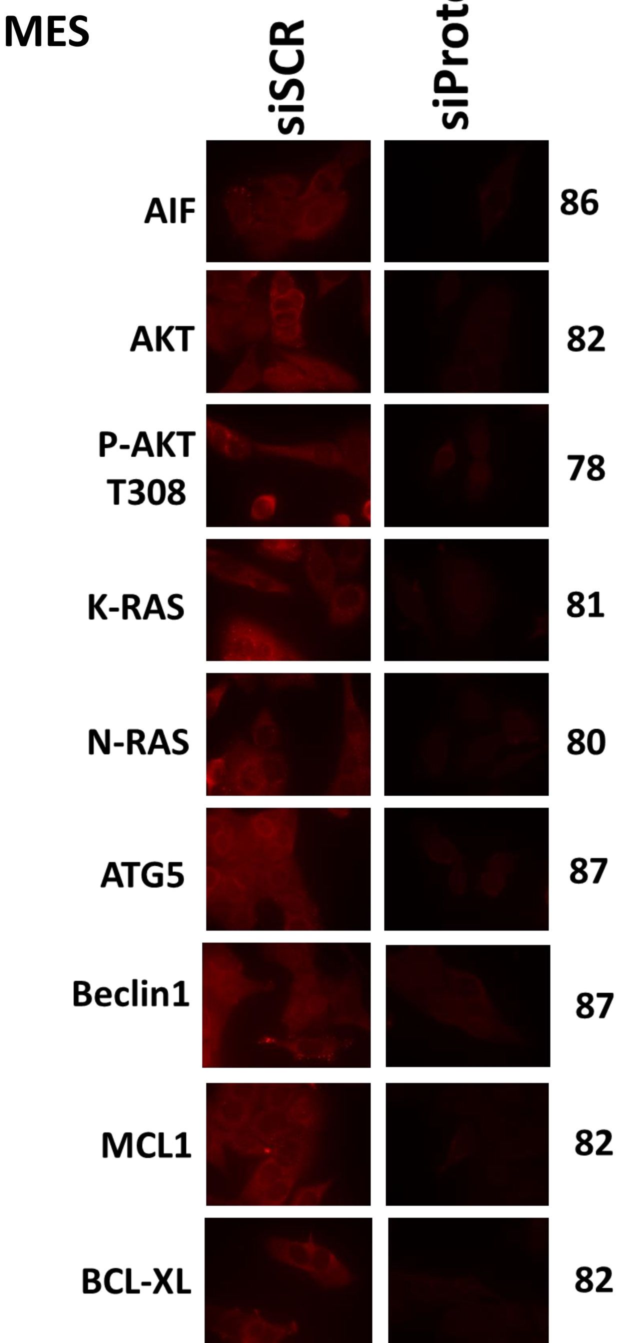

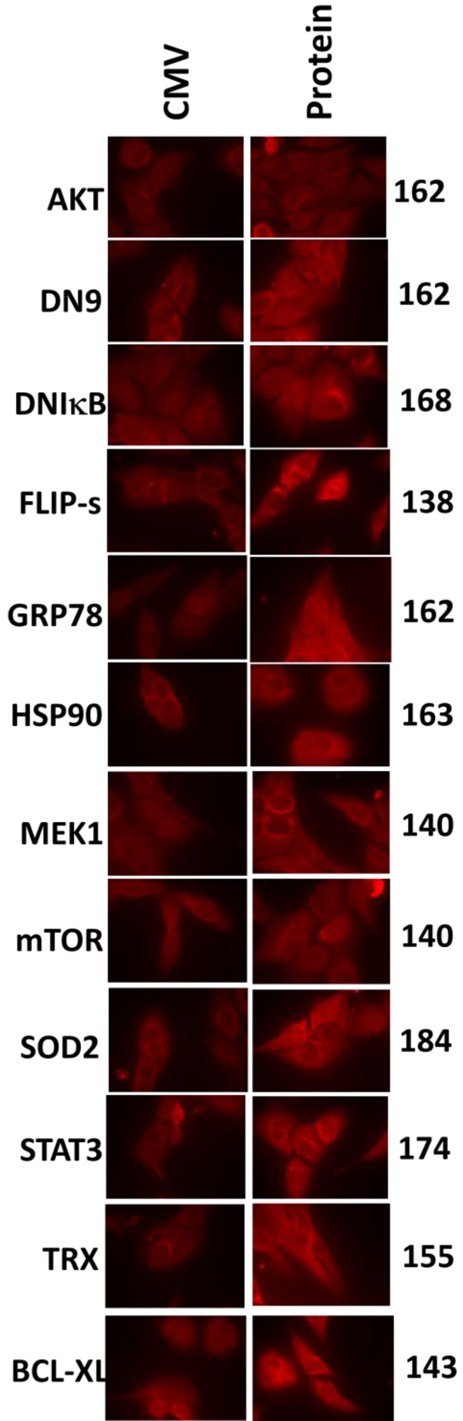

Supplemental Figure 4

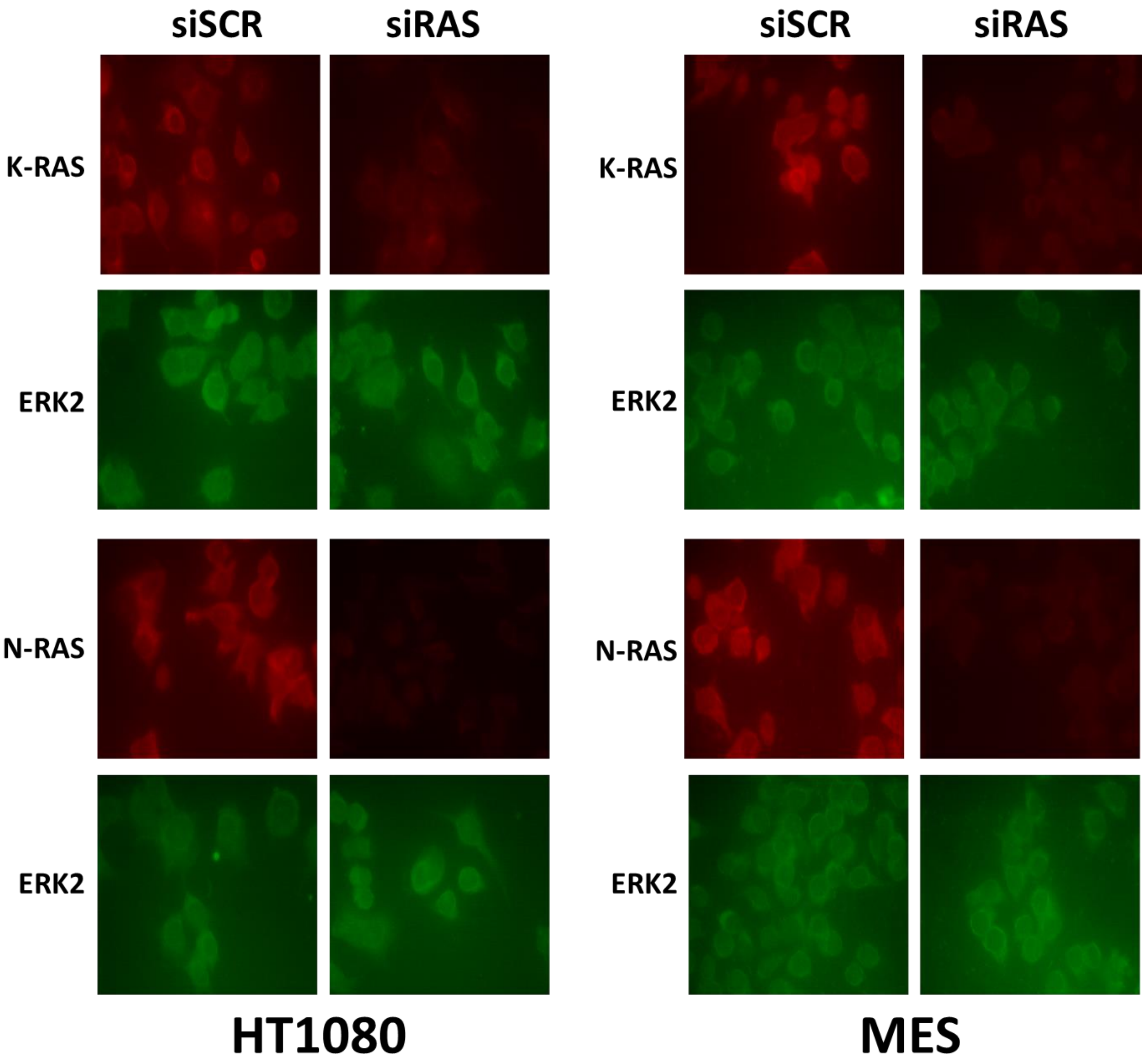

Supplemental Figure 5

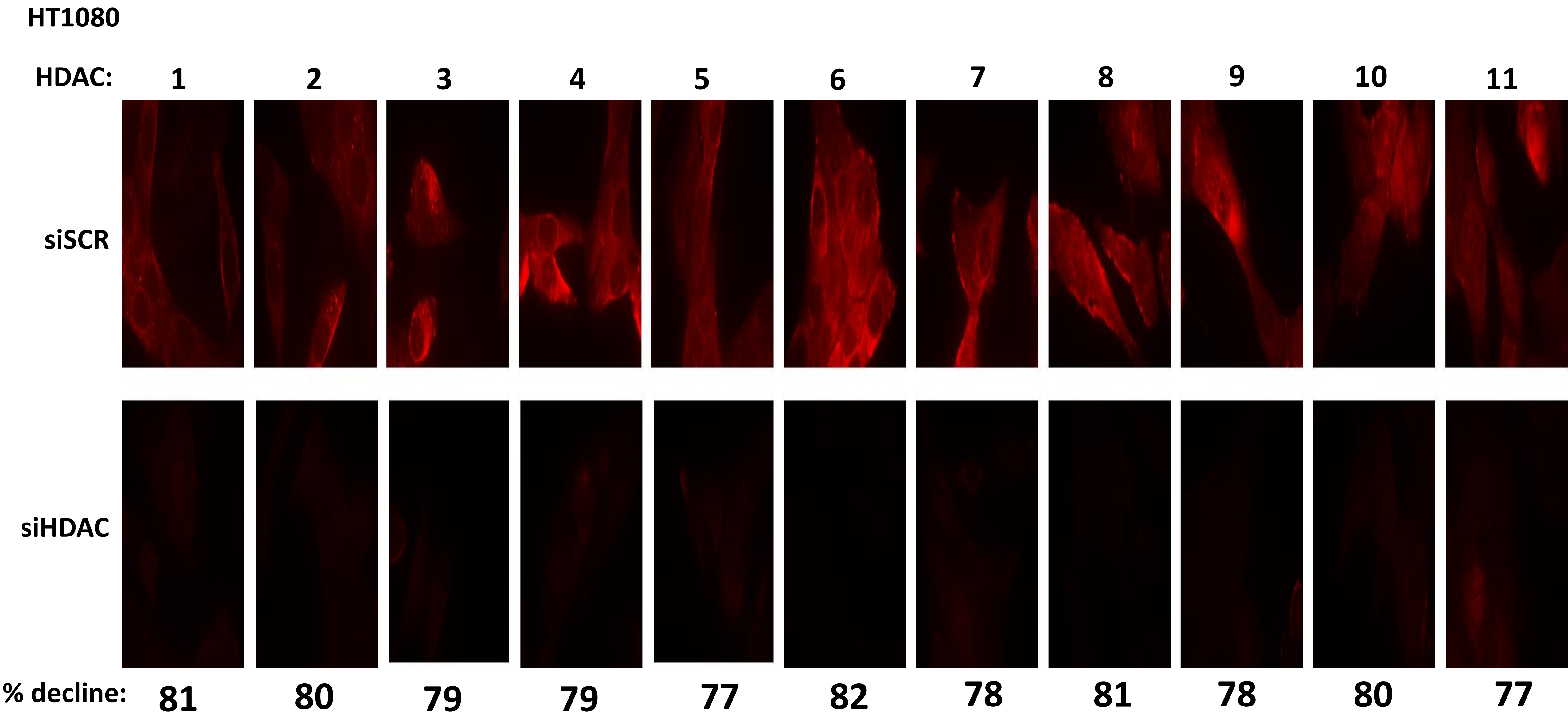

Supplemental Figure 6

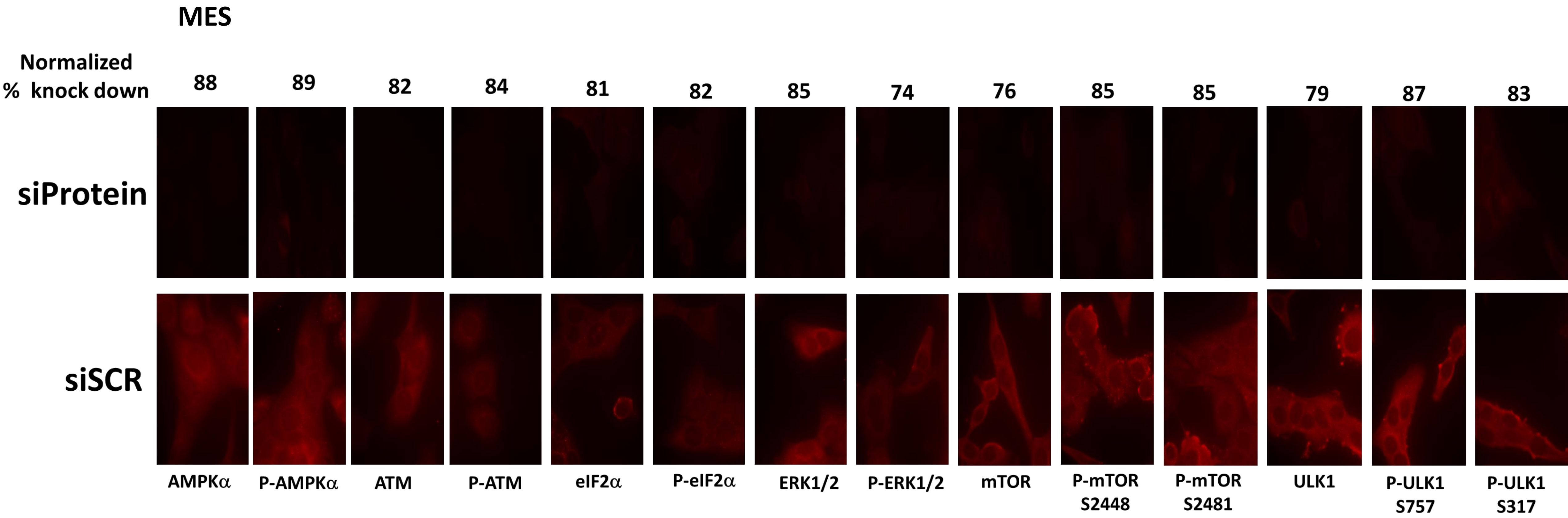

Supplemental Figure 7

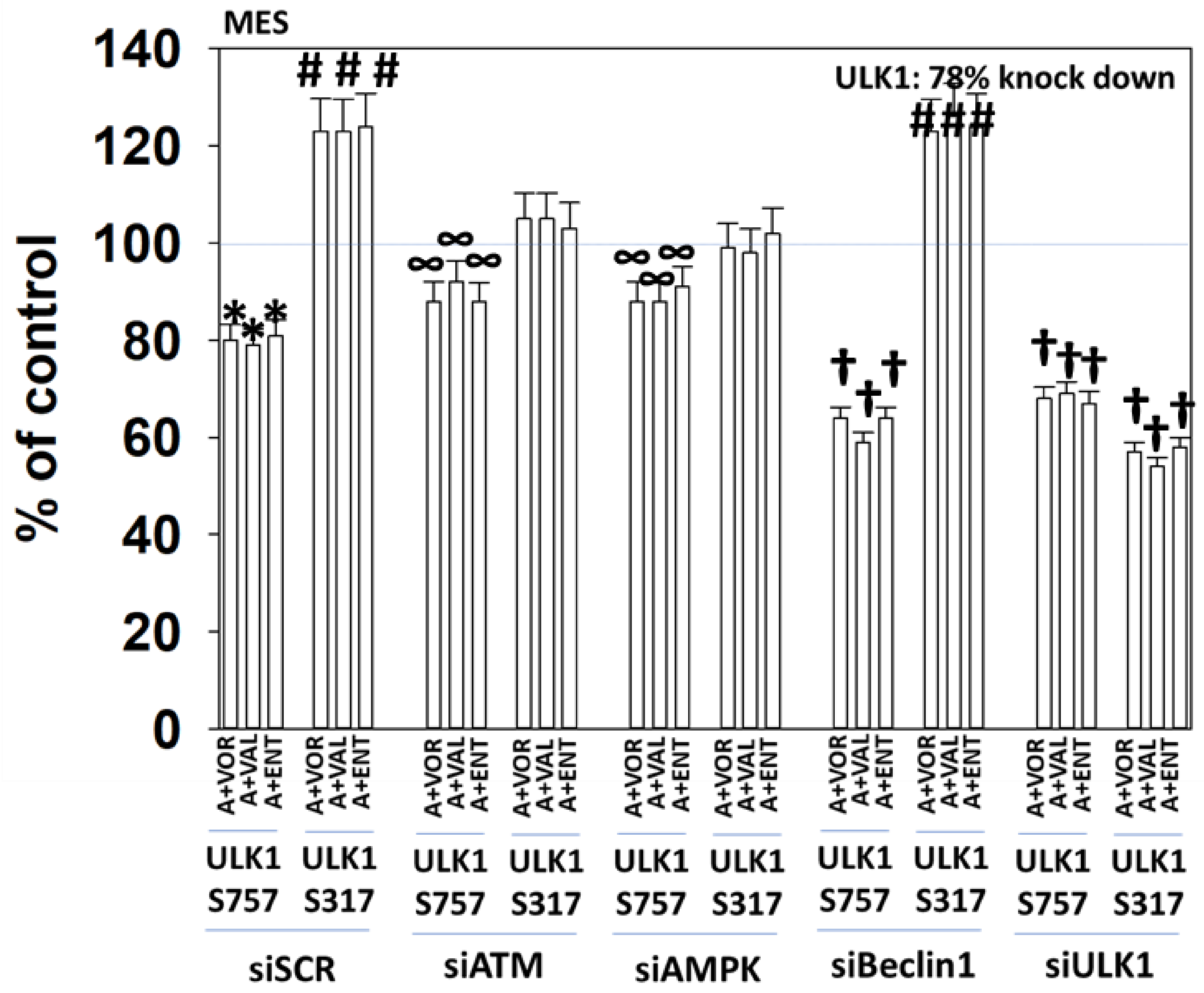

Supplemental Figure 8

HT1080

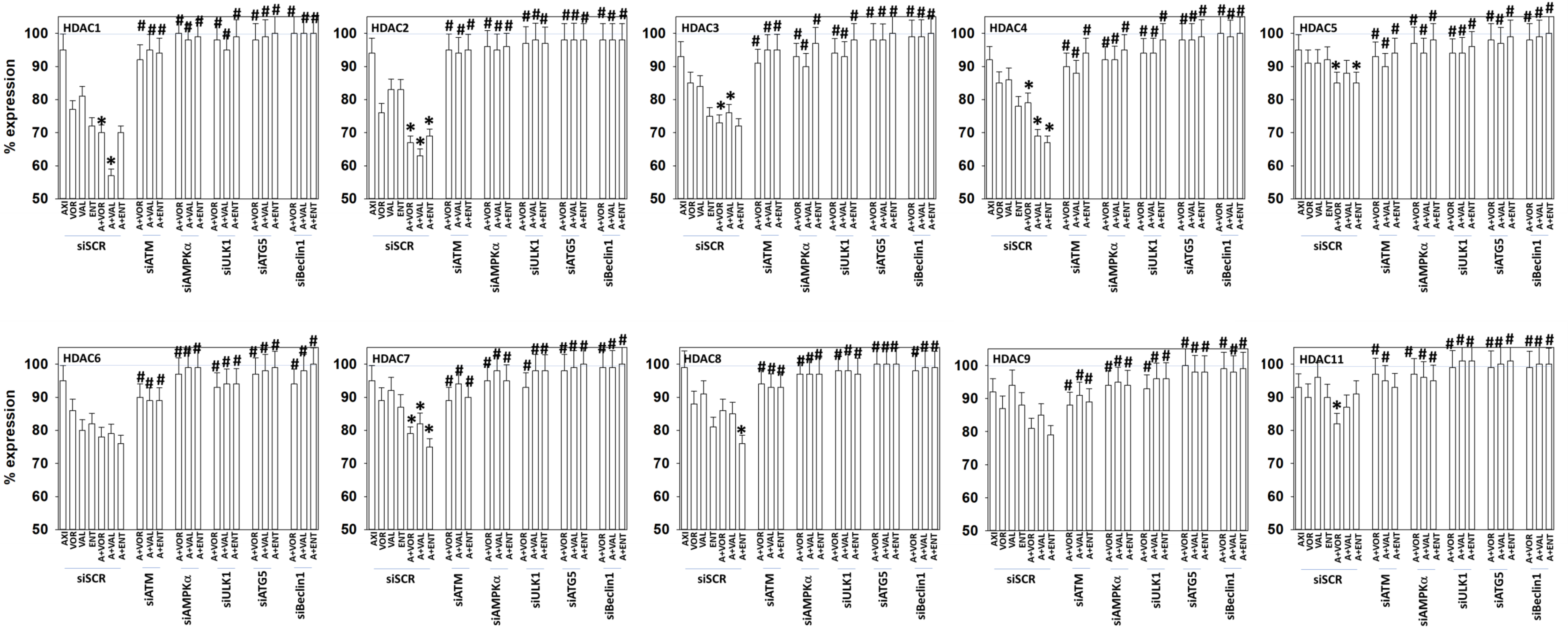

Supplemental Figure 9

MES

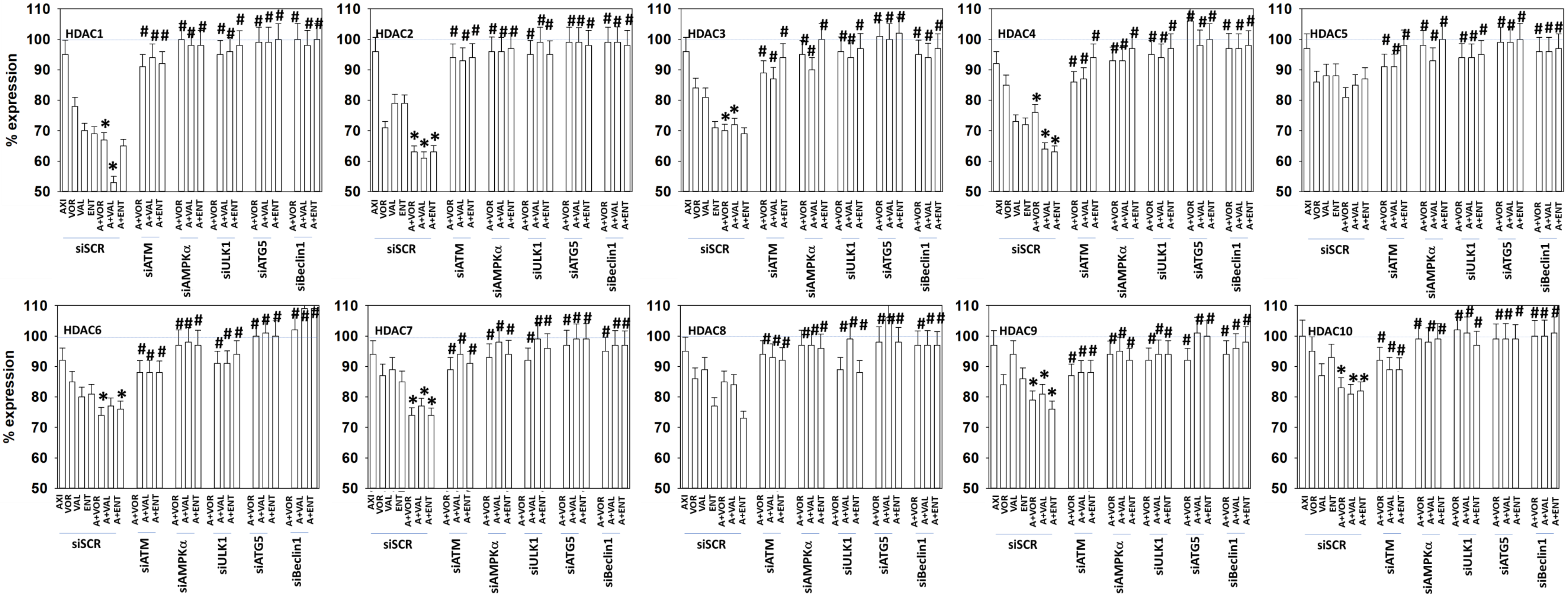

Supplemental Figure 10

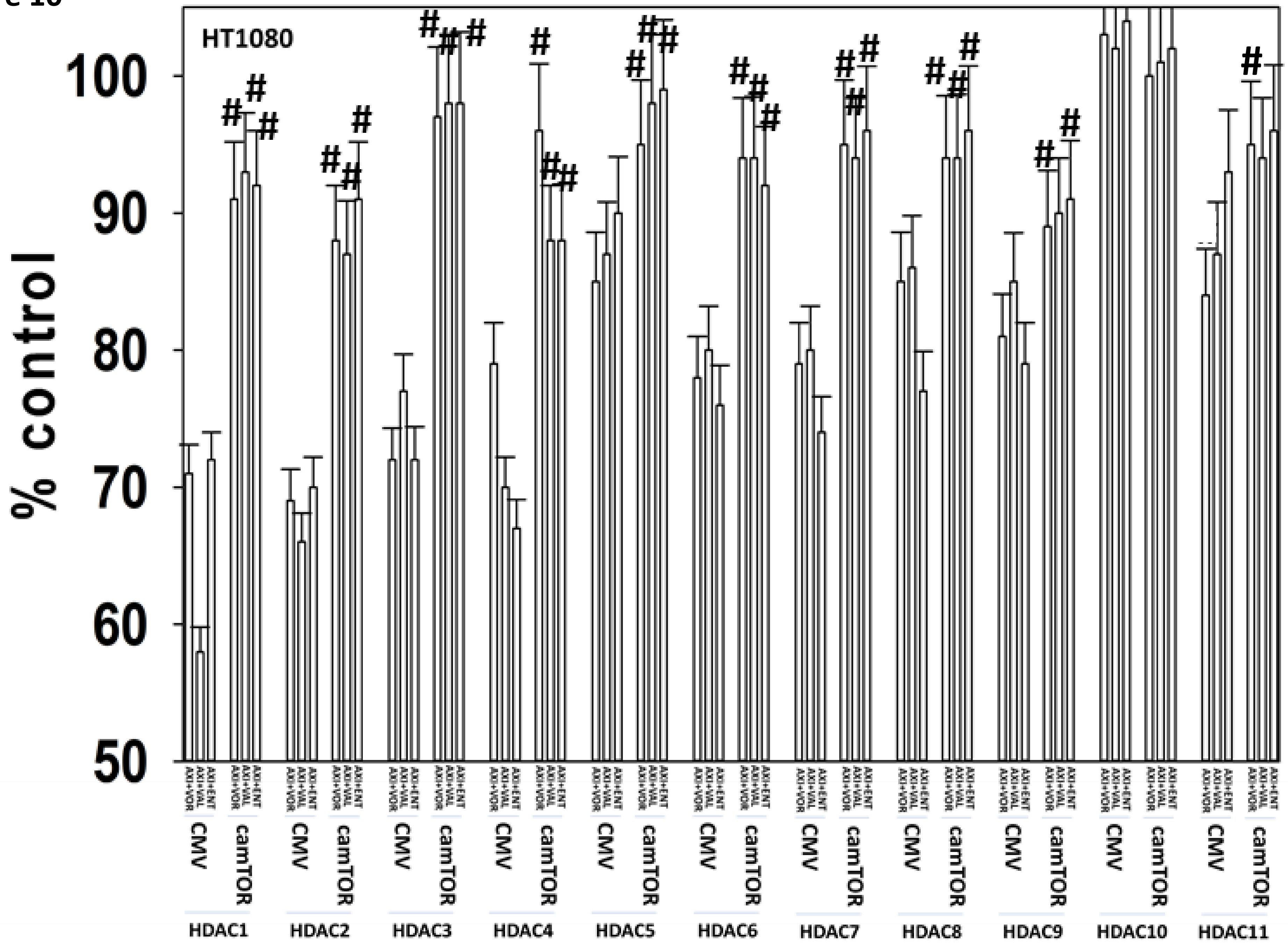

Supplemental Figure 11

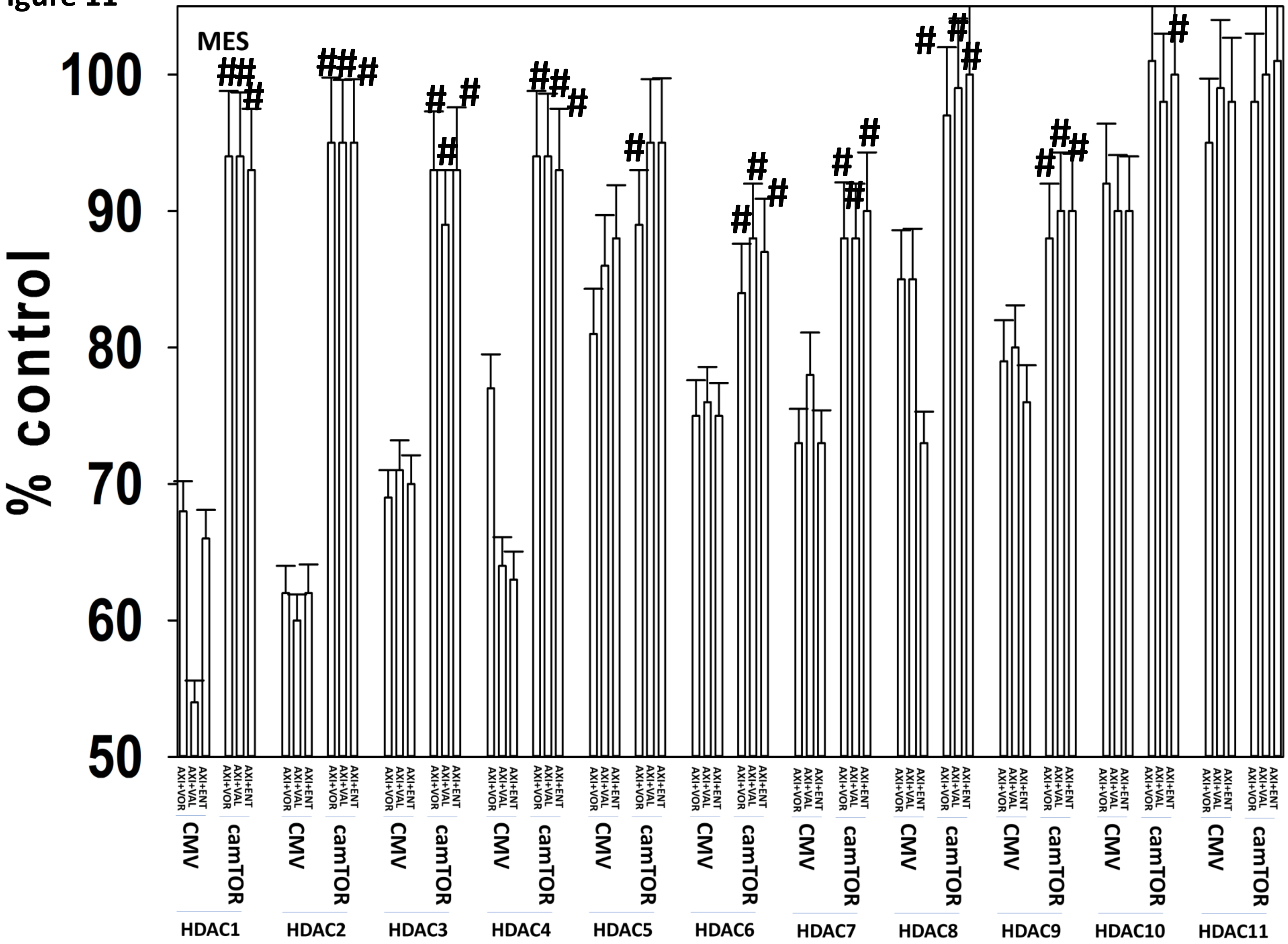

Supplemental Figure 12

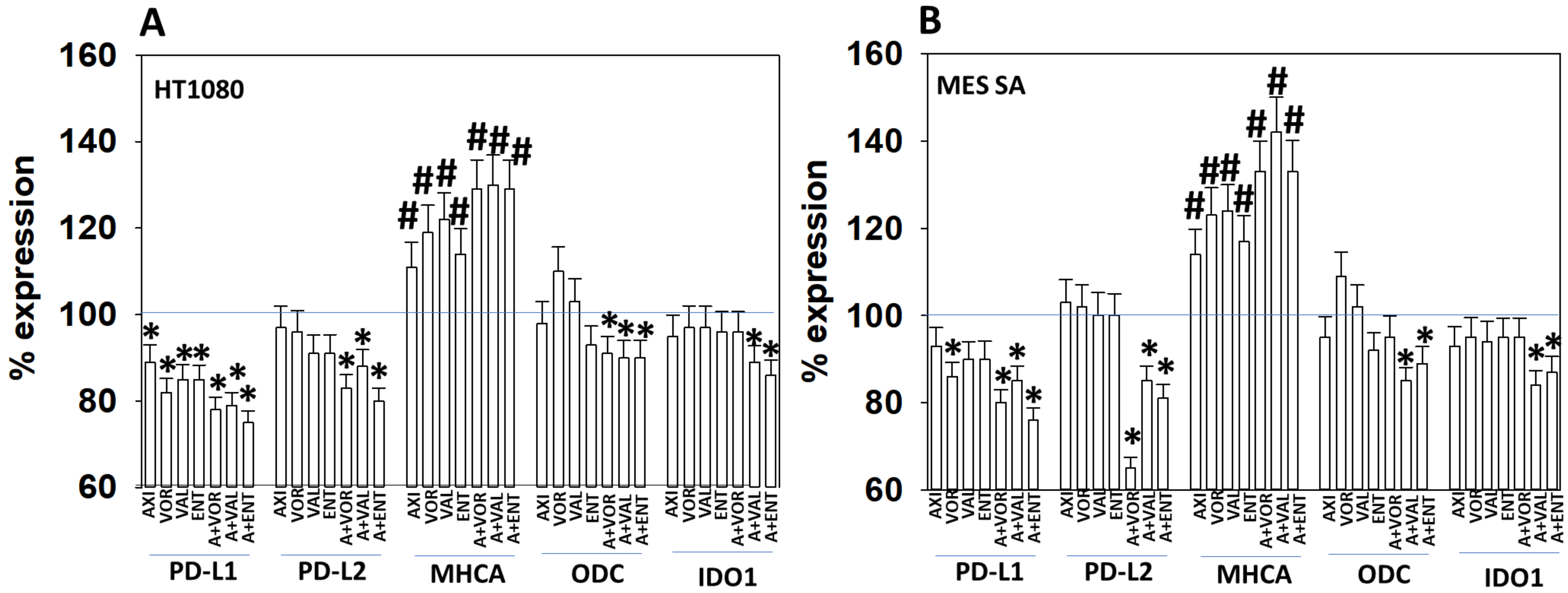

Supplemental Figure 13

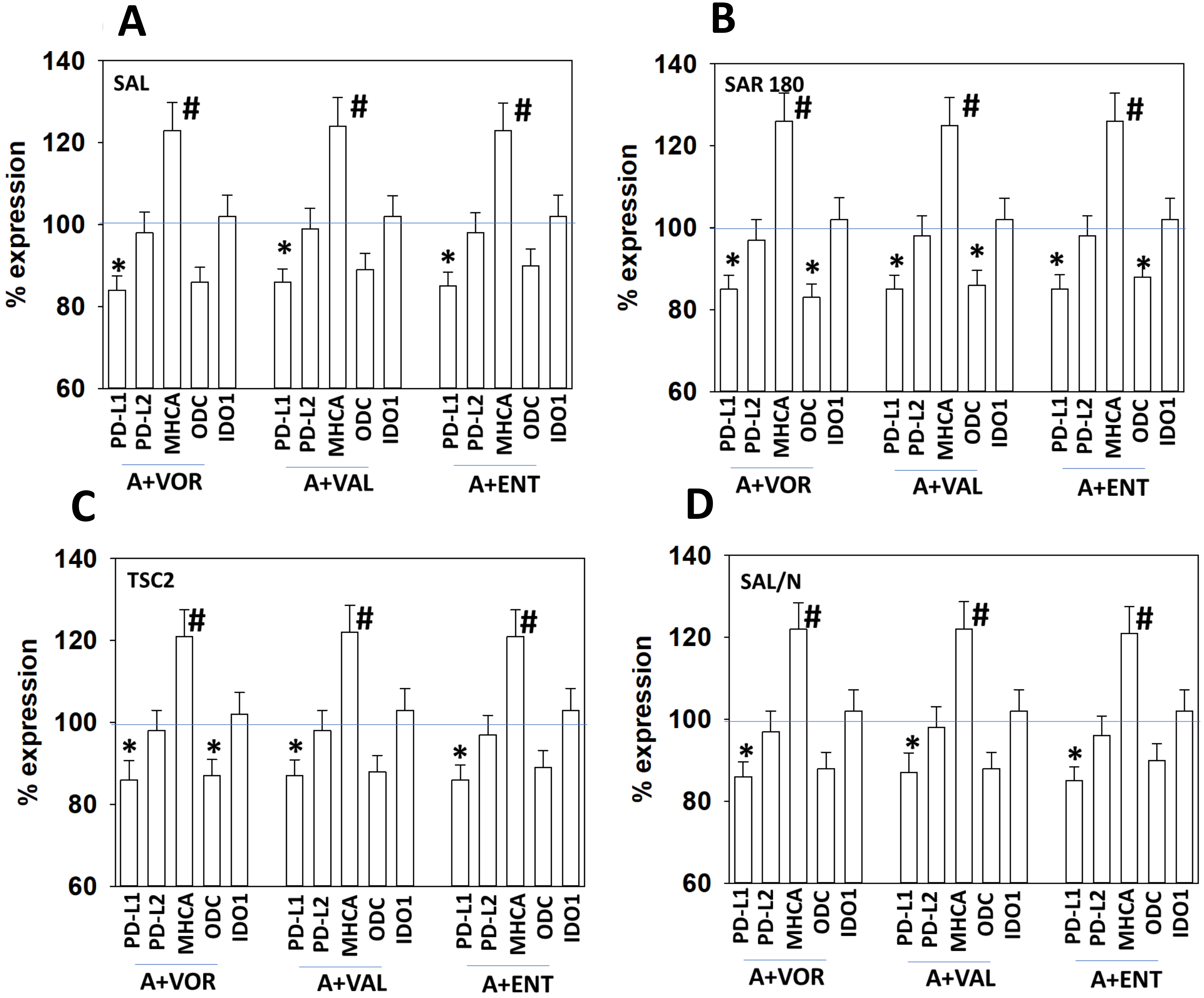

Supplemental Figure 14

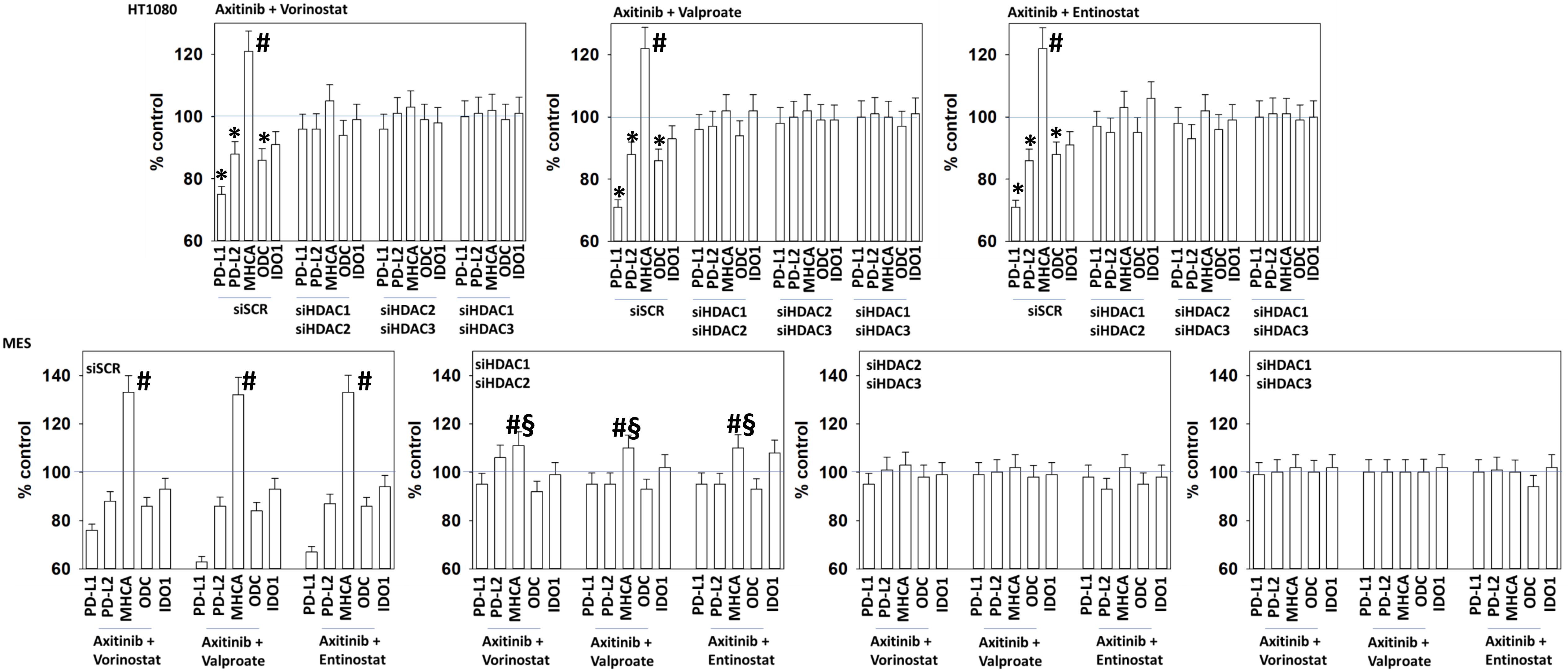

Supplement: Supplementary file 1 [file DataSheet_1.pdf]
